# Targeting Peripheral T-Cell Lymphomas with Chimeric Antigen Receptor Therapy: Mechanistic Challenges, Clinical Landscape and Future Engineering Perspectives

**DOI:** 10.3390/cancers18203400

**Published:** 2026-10-21

**Authors:** Ruxandra Irimia, Pier Paolo Piccaluga

**Affiliations:** 1 “Agrippa Ionescu” Emergency Hospital, 1 Arh. Ion Mincu Street, 077015 Bucharest, Romania; ruxandramariarimia@gmail.com; 2 Biobank of Research, IRCCS Azienda Ospedaliero-Universitaria di Bologna, Via Massarenti, 9, 40138 Bologna, Italy; 3 Department of Medical and Surgical Sciences, Institute of Hematology and Medical Oncology “L&A Seràgnoli”, Bologna University School of Medicine, University of Bologna, Via Massarenti, 9, 40138 Bologna, Italy

**Keywords:** peripheral T-cell lymphoma, PTCL-NOS, CAR-T, CAR-NK, CD7, CD5, TRBC1, CD30, fratricide, genome editing, tumor microenvironment

## Abstract

Peripheral T-cell lymphomas (PTCL) comprise a biologically diverse and clinically aggressive family of mature T/NK-cell neoplasms, for which outcomes remain poor in the majority of patients treated with conventional anthracycline-based frontline regimens. Contemporary classifications (WHO-HAEM5 and ICC) increasingly incorporate immunophenotypic and molecular information, underscoring that “PTCL-NOS” is not a single disease but rather a diagnostic umbrella that includes biologically distinct entities and molecular subgroups. Among these are the TBX21- and GATA3-driven PTCL-NOS subsets, the latter associated with inferior survival and a distinctive cytokine/microenvironmental program. These advances, many of which were shaped by long-standing contributions from the Bologna group, support a rational shift from empiric chemotherapy toward mechanism-based and immune-based interventions. CAR-engineered cellular therapies have revolutionized the care of B-cell malignancies, yet translation to PTCL has been delayed by lineage-specific barriers: (i) *fratricide* during manufacturing when targeting shared T-cell antigens (e.g., CD5, CD7), (ii) the risk of profound and prolonged *T-cell aplasia* (a markedly less “replaceable” toxicity than B-cell aplasia), and (iii) *product contamination* by malignant T-cells in autologous platforms. Recent progress in genome editing (CRISPR/Cas9 and base editing), intracellular antigen “blocking” strategies, and alternative effectors (allogeneic “universal” CAR-T, CAR-NK, and iPSC-derived platforms) has begun to address these obstacles at the manufacturing level, while their downstream clinical consequences (e.g., prolonged cytopenias, infectious risk and delayed immune reconstitution) remain incompletely resolved. Early-phase clinical trials now demonstrate clinically meaningful responses for select targets, particularly CD7, CD5, TRBC1 and CD30, for the pan-T targets. However, the strongest efficacy data remain largely derived from T-cell lymphoblastic malignancies (T-ALL/T-LBL), whereas evidence in mature PTCL remains limited and at an earlier stage, with target-specific differences in safety, immune reconstitution and relapse patterns. This review synthesizes the biological rationale for antigen selection in PTCL, the emerging clinical evidence for leading CAR targets, and the engineering strategies most likely to improve the therapeutic index. It also proposes an integrated hematology perspective on how CAR-based therapies might be positioned relative to transplant and evolving targeted agents in relapsed/refractory PTCL.

## Simple Summary

Peripheral T-cell lymphomas are aggressive forms of cancers caused by mature T lymphocytes. These cancers respond poorly to standard chemotherapy, leaving a pressing need for new treatments. Chimeric antigen receptor (CAR) cell therapy, which reprograms immune cells to recognize and kill tumor cells, has transformed the treatment of B-cell cancers, but its use against T-cell tumors has been held back by problems unique to T-cell lineage: the engineered cells tend to attack one another, healthy T-cells can be destroyed alongside the cancer, and tumor cells can contaminate the therapeutic product. This review summarizes how newer engineering strategies address these obstacles, examines which target molecules show the most promise in early clinical trials, and argues that the choice of target should be guided by the molecular subtype of each tumor. Throughout, we make explicit where the current evidence is solid and where it remains preliminary.

## 1. Introduction

Effective treatment of peripheral T-cell lymphomas (PTCLs) remains a major clinical challenge. Conventional anthracycline-based chemotherapy regimens, followed by autologous stem cell transplantation in selected cases, is associated with high rates of relapse or primary refractory disease [1]. The prognosis of chemorefractory, transplant-ineligible disease is dismal: after relapse without transplantation, median overall survival is approximately 5.5 months [2]. In contrast, relapsed transplant-eligible patients fare considerably better prognosis (2-year overall survival approximately 34% [3]); therefore, the population for which CAR-T therapy is presently intended is specifically the refractory, transplant-ineligible group [1].

While the introduction of chimeric antigen receptor (CAR) T-cell therapy has fundamentally changed the treatment paradigm for B-cell malignancies, the application of this technology to T-cell-derived neoplasms has been significantly delayed by biological hurdles unique to the T-lineage. Unlike B-cell targeting, where the depletion of the healthy B-cell compartment is a manageable clinical toxicity, the targeting of pan-T-cell antigens risks profound immunosuppression, life-threatening fratricide among the therapeutic cells themselves, and the potential for contaminating the final product with malignant clones [4,5]. Recent advances in genomic engineering, particularly the use of CRISPR/Cas9-mediated gene disruption and the exploration of alternative effector cells such as natural killer (NK) cells, have provided the technical foundation necessary to circumvent these obstacles [6,7]. In this article the authors seek to provide an exhaustive analysis of the current landscape of CAR-directed therapies in PTCL, examining the biological rationale for specific antigen selection, the clinical outcomes of ongoing trials, and the emerging perspectives on novel targets designed to optimize the therapeutic index. Several reviews have surveyed cellular immunotherapy in T-cell malignancies; the present work is deliberately narrower and complementary. Rather than cataloging constructs, we organize antigen selection around the molecular subclassification of PTCL—the GATA3- and TBX21-defined subsets of PTCL-NOS and the transcriptional programs of AITL, ALCL and ENKTL—and treat the resulting target-to-subtype mapping as an explicit, testable framework rather than established practice. To our knowledge no efficacy dataset to date is stratified by molecular subtype; we therefore frame this integration as a research agenda, stating throughout where rationale ends and evidence begins.

### 1.1. Molecular Pathogenesis and the Rationale for Cellular Immunotherapy

**Figure 1:**
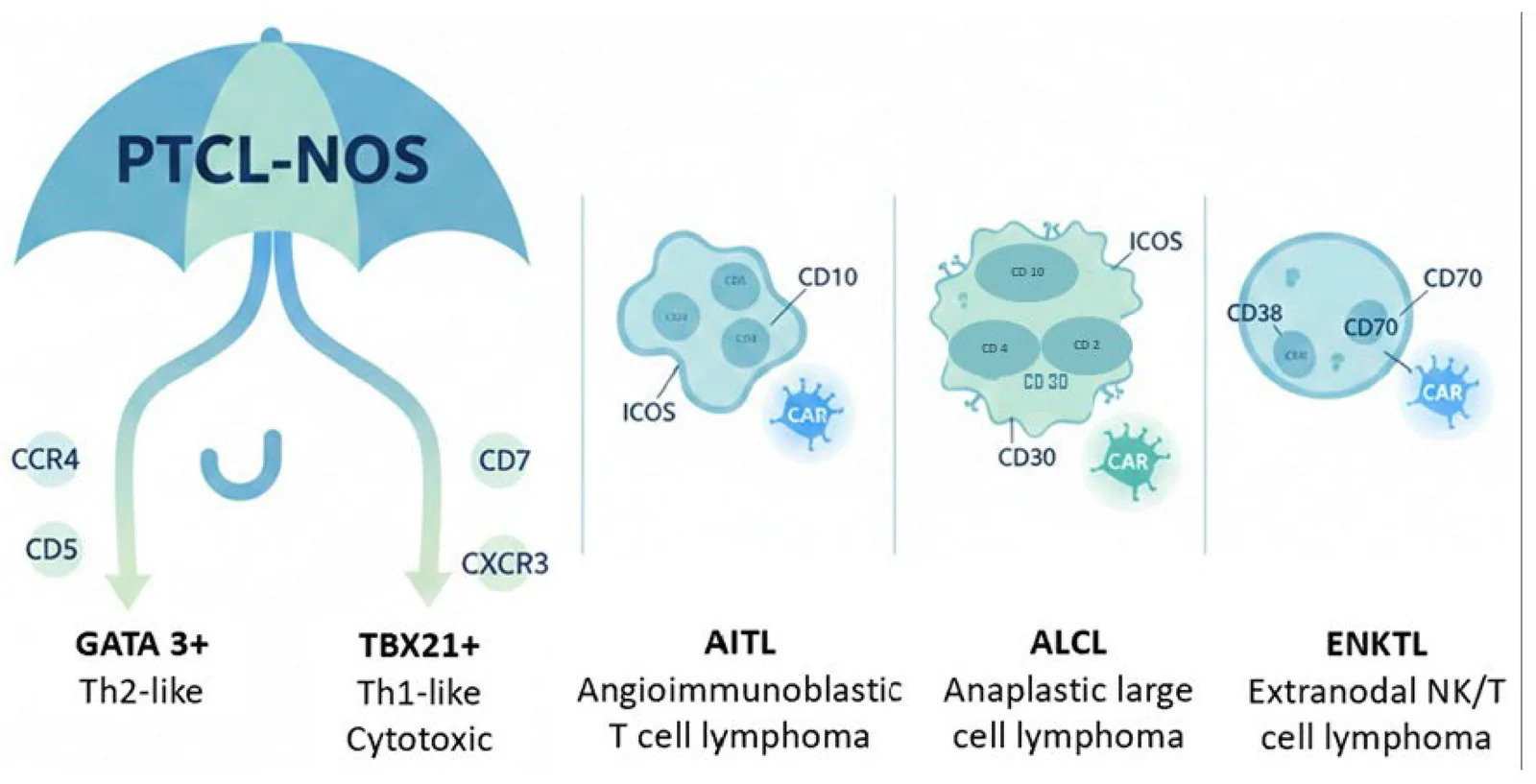
Molecular subtypes of PTCL and rational antigen selection. Note the biological diversity of PTCL, highlighted by the transition from the “PTCL-NOS” diagnostic umbrella to molecularly defined subgroups. Common surface antigens (CD7, CD5, CD30, CCR4, etc.) are mapped onto specific clinical entities such as GATA3+ and TBX21+ PTCL-NOS, AITL, and ALCL, providing a visual roadmap for targeted immunotherapy.

Peripheral T-cell lymphomas are a diverse group of non-Hodgkin lymphomas (NHL) arising from mature, post-thymic T-lymphocytes or NK-cells (Figure 1). They account for approximately 10% to 15% of all NHL cases globally, though their incidence exhibits significant geographic variation, being more prevalent in parts of Asia and South America [8,9]. The 2022 World Health Organization (WHO) and International Consensus Classification (ICC) systems recognize over 30 distinct subtypes, including peripheral T-cell lymphoma not otherwise specified (PTCL-NOS), angioimmunoblastic T-cell lymphoma (AITL), anaplastic large-cell lymphoma (ALCL), and extranodal NK/T-cell lymphoma (ENKTL) [8,9].

The biological heterogeneity of these malignancies is matched by a complex mutational landscape [10]. Many PTCL subtypes, particularly those of follicular T-helper (Tfh) cell origin like AITL, are driven by recurrent mutations in epigenetic regulators such as *TET2*, *DNMT3A*, and *IDH2*, alongside the *RHOA*^G17V^ mutation [9,11]. These alterations contribute to a highly immunosuppressive tumor microenvironment (TME) characterized by an abundance of regulatory T-cells (Tregs) and tumor-associated macrophages (TAMs), which physically and chemically shield malignant cells from immune surveillance [12]. Conventional therapies, such as CHOP (cyclophosphamide, doxorubicin, vincristine, and prednisone), fail to provide durable remissions in the majority of patients, with five-year overall survival (OS) rates often remaining below 30% [13,14]. Consequently, there is an urgent clinical mandate for targeted immunotherapies that can bypass the chemoresistance mechanisms inherent in these aggressive clones.

### 1.2. Antigenic Heterogeneity and Subtyping as a Guide for CAR Selection

The identification of suitable targets in PTCL is complicated by the fact that malignant T-cells often retain the surface markers of their healthy counterparts [15,16]. PTCL-NOS, for instance, is frequently subdivided into molecular subgroups based on the expression of master transcription factors: the PTCL-GATA3 subgroup, which exhibits a Th2-like signature and is associated with a poor prognosis, and the PTCL-TBX21 subgroup, which displays a Th1/cytotoxic profile [17,18,19]. These subtypes not only differ in their clinical behavior but also in their potential targetable antigens (Table 1). The PTCL-GATA3 subtype is often associated with the expression of CCR4, whereas the TBX21 subtype may express higher levels of cytotoxic markers [15].

### 1.3. Biological Impediments: Fratricide and T-Cell Aplasia

The most significant barrier to the development of CAR-T-cells for T-cell malignancies is “fratricide,” a phenomenon where the therapeutic T-cells destroy each other due to a natural expression of the same target antigen [4]. When T-cells are engineered to express a CAR against a pan-T marker such as CD7, the CAR-T-cells immediately recognize the CD7 molecules on neighboring T-cells, leading to their mutual destruction during the manufacturing process. This self-targeting results in poor cell expansion and significantly reduced viability of the final product [20].

Beyond manufacturing difficulties, the clinical use of pan-T-cell-directed CARs leads to profound T-cell aplasia [5]. Unlike B-cell aplasia, which can be managed with intravenous immunoglobulin (IVIG) replacement, the loss of the T-cell compartment results in a state of severe immunodeficiency similar to that seen in advanced HIV/AIDS or following intensive myeloablative conditioning [5,21]. This necessitates a high degree of clinical vigilance for opportunistic infections, including cytomegalovirus (CMV), Epstein–Barr virus (EBV), and fungal pathogens [22].

**Figure 2:**
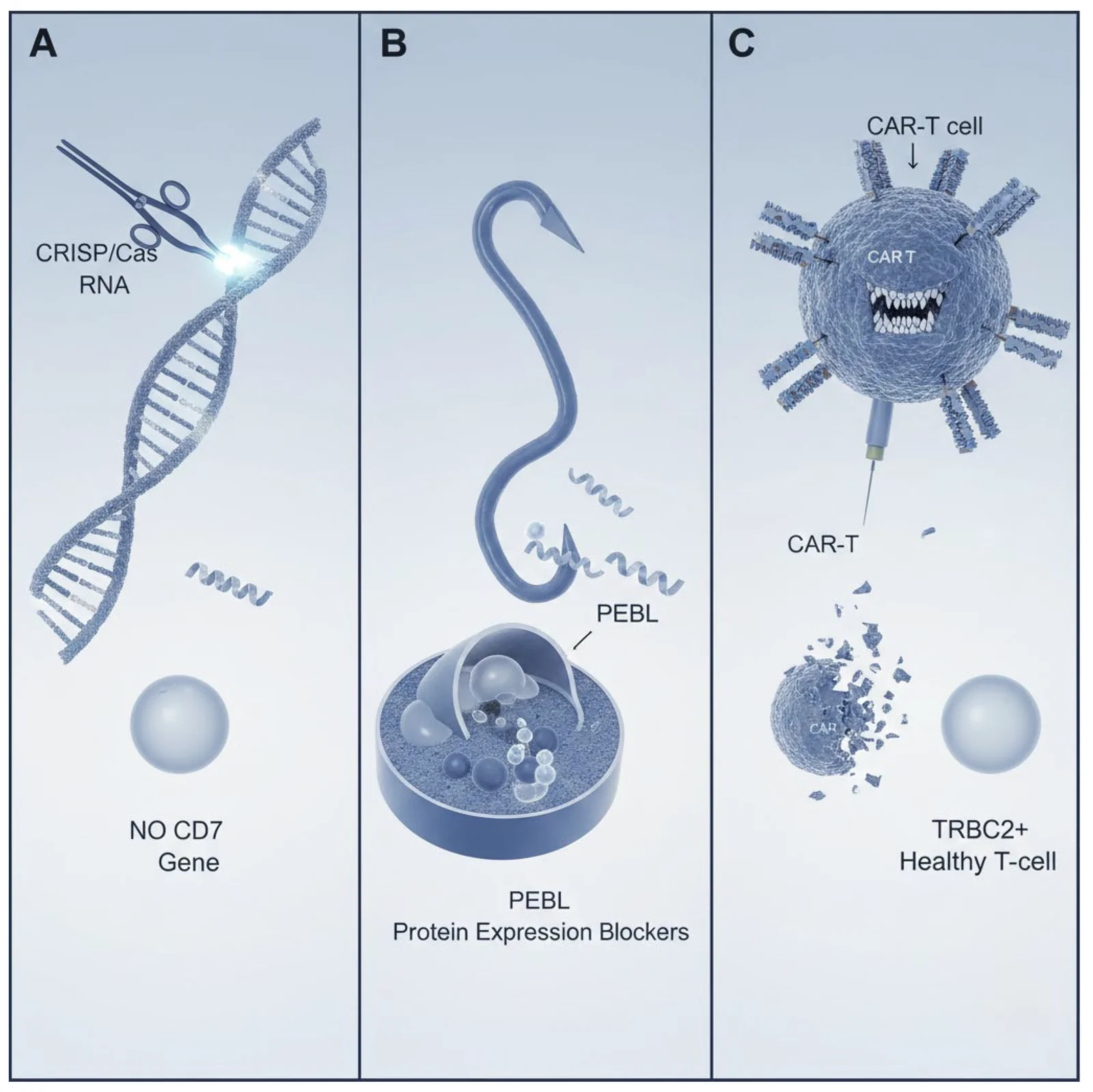
Strategies to overcome fratricide and T-cell aplasia. A schematic representation of the three primary engineering techniques used to enable CAR-T production for T-cell malignancies: (**A**) genomic disruption via CRISPR/Cas9 to knockout the target antigen (e.g., CD7); (**B**) protein expression blockers (PEBL) that sequester antigens in the ER/Golgi; and (**C**) clonal-specific targeting of the TRBC1 constant domain, which spares the healthy TRBC2+ T-cell compartment.

To overcome fratricide, researchers have developed several sophisticated engineering strategies. The most prominent involves the genomic disruption of the target antigen in the CAR-T-cells themselves (Figure 2). By using CRISPR/Cas9 or base editing to knock out the gene encoding the target (e.g., CD7), the CAR-T-cells become resistant to self-recognition [4]. This approach has been successfully validated in preclinical models and is now a standard component of many clinical-stage CD7 CAR-T products [20].

Alternative methods include the use of protein expression blockers (PEBL), which utilize a single-chain variable fragment (scFv) linked to an endoplasmic reticulum or Golgi retention signal. This system captures the target protein within the secretory pathway, preventing its translocation to the cell surface [22]. Additionally, the use of naturally CD7-negative or CD5-negative T-cells has been explored, though these populations are rare and may lack the necessary functional diversity for a robust anti-tumor response [4].

Whereas fratricide is a manufacturing problem with mature solutions, T-cell aplasia is a clinical problem that remains substantially unsolved, and several mitigation strategies are under investigation. Antigen-sparing designs aim to preserve a functional T-cell compartment: clonal-restricted targeting of TRBC1 (Section 1.6) spares the TRBC2 repertoire, and subset-restricted targets such as CD37, ROR1 and CCR8 (Section 1.9) avoid pan-T depletion by construction. Time-limited CAR activity is a second strategy, previously validated in B-cell-targeted cellular therapies. Transient mRNA-based CARs, drug-regulated “on-switch” constructs and incorporated safety switches such as inducible caspase-9 [23,24] allow the engineered population to be curtailed once tumor control is achieved. Third, consolidative allogeneic HSCT is often pursued as a necessity in order to reconstitute immunity after deliberate aplasia (Section 2.5). Finally, CAR-NK and other non-T effectors (Section 1.8) sidestep the problem by not requiring depletion of the host T-cell compartment. None of these is yet a definitive answer, and the durability-versus-aplasia trade-off—deep, lasting responses tend to require deep, lasting T-cell depletion—remains the central unresolved tension of the field.

### 1.4. CD7-Targeted CAR-T Therapy: The Clinical Vanguard

CD7 is a 40 kDa transmembrane glycoprotein expressed on the majority of T-cell acute lymphoblastic leukemia (T-ALL) cases. In mature nodal PTCL, by contrast, CD7 is frequently downregulated or lost, with retained expression confined to a subset of cases [25,26]. This dissociation between lymphoblastic and mature disease is clinically consequential: because CAR-mediated cytotoxicity depends on antigen density, low or heterogeneous CD7 expression represents both a patient-selection criterion and a plausible route to antigen-negative escape (see Section 2). CD7 targeting is therefore most robust in CD7-retained disease, and CD7 status should arguably guide eligibility rather than be presumed. As CD7 is dispensable for normal T-cell development and function, several engineering strategies have been developed to prevent its expression on CAR-T-cells and avoid fratricide, including CRISPR–Cas9-mediated gene disruption and intracellular retention systems through protein expression blockers [27,28]. Clinical trials of CD7 CAR-T-cells have demonstrated some of the most impressive response rates observed in the PTCL field to date [27].

**Figure 3:**
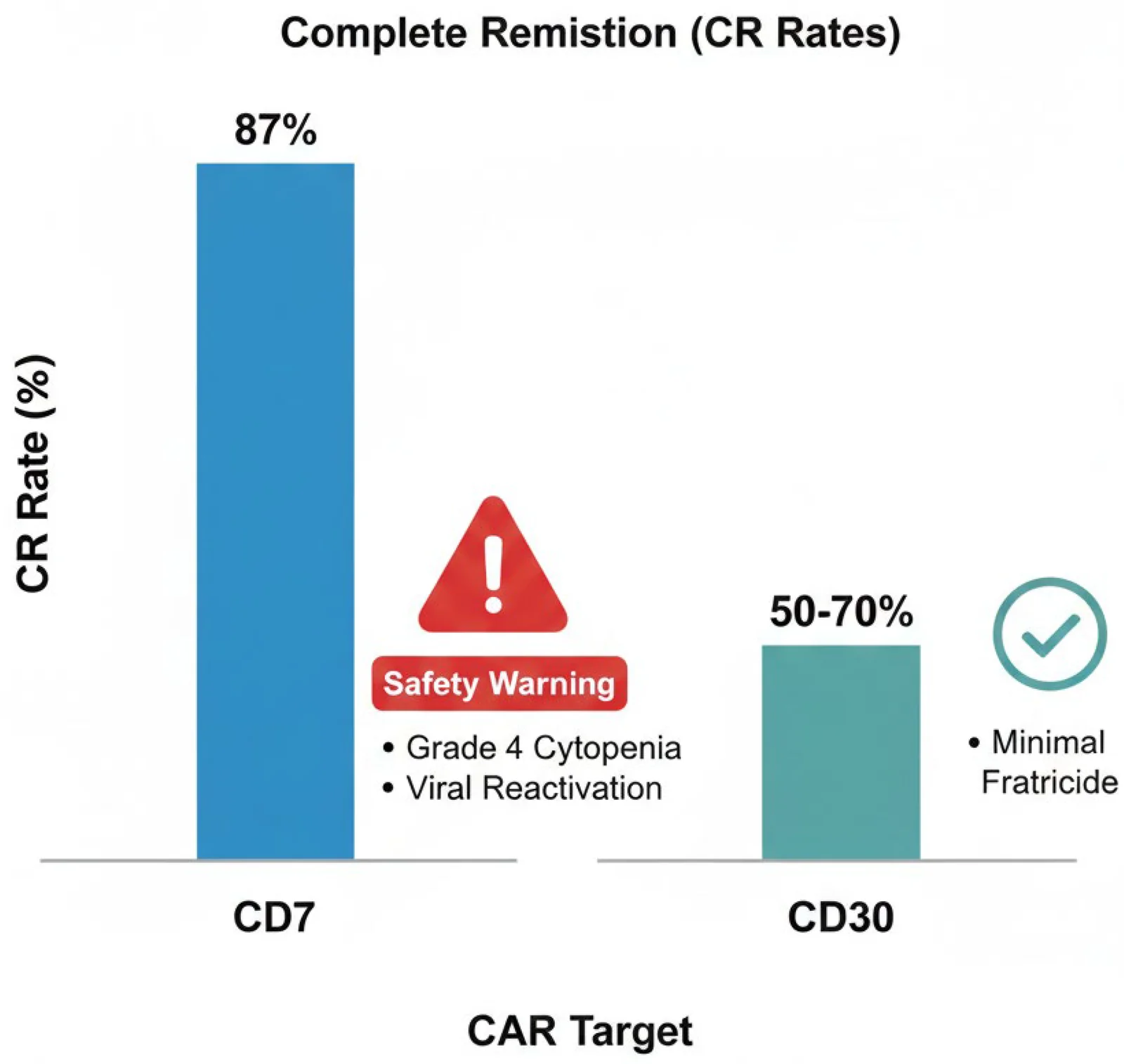
Clinical efficacy and safety profile of leading CAR targets. The image synthesizes recent trial data, showing the high complete remission (CR) rates achieved with CD7-targeted therapies (blue) (often used as a bridge-to-transplant) and the established safety of CD30-targeted CARs (green) in ALCL. The chart highlights the trade-off between the profound hematologic toxicity of pan-T targets and the narrow but safer profile of CD30.

The most mature clinical experience with CD7 CAR-T derives predominantly from lymphoblastic rather than mature disease, and this distinction must be kept explicit. A systematic review and meta-analysis of 13 studies (200 patients) reported pooled complete remission rates approaching 87% (Figure 3); that cohort, however, comprised CD7-positive hematologic malignancies that were overwhelmingly T-ALL/T-LBL and did not report a mature-PTCL subgroup, so a PTCL-specific complete remission rate cannot be derived from it [29]. One notable product, RD13-01, an allogeneic universal CD7 CAR-T-cell therapy, utilized CRISPR/Cas9 to knock out CD7, TRAC, and RFX5 (Table 2). In a phase I trial enrolling a mixed CD7-positive population that included PTCL-NOS, T-ALL and AML, the overall response rate was 63.6%, with several durable remissions beyond ten months; the number of mature-PTCL patients and their disaggregated outcomes were, however, limited. Interpreted stringently, the evidence base for CD7 CAR-T in mature PTCL remains early and numerically small, and the field’s headline response rates are driven by lymphoblastic disease [30].

However, the safety profile of CD7-targeted therapy is complex. While the occurrence of severe cytokine release syndrome (CRS) and neurotoxicity (ICANS) is relatively low (12% and 4%, respectively), hematologic toxicity is profound [29]. Most patients developed prolonged grade 4 neutropenia and lymphopenia [22,27]. Graft versus host disease, bacterial and fungal infections as well as viral reactivation are also significant risks [27,30]. Additionally, in the RD13-01 trial, one patient succumbed to EBV-related diffuse large B-cell lymphoma (DLBCL) following CAR-T infusion, highlighting the risks associated with prolonged T-cell and NK-cell depletion [30].

The consensus among investigators is that CD7 CAR-T therapy often serves as a highly effective “bridge-to-transplant.” Consolidative allogeneic hematopoietic stem cell transplantation (allo-HSCT) is frequently employed once CR is achieved to restore the immune system and prevent relapse from CD7-negative clones [15].

### 1.5. CD5-Targeted CAR-T Therapy: Modulating TCR Signaling

CD5 is expressed on approximately 85% of T-cell malignancies and functions as a negative regulator of T-cell receptor (TCR) signaling, contributing to the maintenance of immune tolerance [4,33]. Unlike CD7, the internalization of CD5 upon CAR engagement appears to be more rapid, which may lead to a more transient and manageable fratricide effect [20].

In a phase I trial (NCT0308190) evaluating autologous CD5 CAR-T-cells in 17 patients with relapsed/refractory (r/r) T-cell lymphomas, the ORR was 44%, with complete responses observed in two patients [34]. Importantly, the trial demonstrated that CD5 CAR-T-cells can be successfully manufactured and administered without the need for genomic disruption of the CD5 gene in every case, though CRISPR-mediated knockout has been shown to improve expansion kinetics in other settings [35]. The toxicities associated with CD5 targeting CAR-Ts were largely restricted to cytopenias, with no cases of severe CRS or ICANS reported, suggesting that CD5 may offer a wider safety margin than CD7 in certain clinical contexts [34].

### 1.6. Selective Domains Targeting of TRBC1

One of the most innovative approaches to avoiding global T-cell aplasia is the selective targeting of the TCR constant domain. The human TCR beta-chain constant domain is encoded by two nearly identical genes, *TRBC1* and *TRBC2* [36]. Each T-cell expresses either *TRBC1* or *TRBC2* [37]. Since T-cell lymphomas are clonal, the entire malignant population will express only one of these domains (approximately 50% are TRBC1-positive), whereas the healthy T-cell compartment will remain a mixture of both [37].

The LibraT1 study (NCT03590574) evaluated AUTO4, a TRBC1-directed autologous CAR-T-cell therapy, in patients with r/r TRBC1-positive PTCL [37]. Preliminary results from the dose-escalation phase showed that AUTO4 was well-tolerated, with only one of ten patients developing grade 3 CRS, which was promptly remitted after Tocilizumab administration [37]. A total of 40% of the evaluable cases achieved complete metabolic response, durable beyond one year in several cases [37]. This strategy allows for the preservation of the TRBC2-positive healthy T-cells, which may provide sufficient immune protection against pathogens and reduce the need for consolidative transplantation [37].

The same logic applies in mirror image to TRBC2-restricted clones, which constitute roughly the other half of cases and are, by definition, not addressed by TRBC1-directed products; dedicated TRBC2-directed constructs would be required for full coverage of the disease but, to our knowledge, remain undeveloped, a genuine gap in the field. Two limitations further temper the strategy. First, its selectivity depends on faithful mutually exclusive constant-region expression; loss or dual expression under selective pressure would permit escape, and durability data remain immature. Second, TRBC1/2 restriction is demonstrable only where clonal constant-region usage is established diagnostically, adding a companion-diagnostic requirement not needed for pan-T targets. TRBC-directed therapy is thus the clearest embodiment of the fratricide-free, aplasia-sparing ideal, yet also the most dependent on precise molecular characterization of the individual tumor, reinforcing the subtype-guided framework argued in Section 1.10.

### 1.7. CD30-Targeted CAR-T Therapy: Success in ALCL and Beyond

CD30, a member of the TNF receptor superfamily, is a hallmark of anaplastic large-cell lymphoma (ALCL) and classical Hodgkin lymphoma (cHL), but its expression is also found in subsets of PTCL-NOS and AITL [9]. Because CD30 expression is minimal on healthy resting T-cells, CD30 CAR-T therapy carries a very low risk of fratricide and systemic immunosuppression [9].

A caveat specific to CD30 deserves emphasis. Across a series of 376 non-cutaneous PTCLs, CD30 expression by immunohistochemistry was heterogeneous both across and within entities, correlating closely with mRNA levels. The expression was bimodally distributed, separating high- from low-expressing cases, with roughly half of non-anaplastic PTCLs positive at a 5% threshold against near-uniform positivity in ALCL [38,39]. Because CAR activity is antigen-density-dependent (Section 2.1), this heterogeneity, dim and partial outside ALCL, plausibly underlies the more modest and less durable responses seen beyond ALCL and constitutes a built-in resistance mechanism, as CD30-low subclones survive and repopulate. Therefore, a simplistic binary definition of tumoral CD30 positivity is insufficient for the broader PTCLs. Quantitative or spatial assessment of CD30, and dual-targeting to cover CD30-low fractions, are more rational therapeutic approaches.

Clinical trials (NCT03049449, NCT02690545) have demonstrated ORRs between 50% and 70% in CD30-positive lymphomas [40,41]. In a phase I study using CD30 CAR-T-cells as consolidation after autologous HSCT for Hodgkin, ALCL, AITL, PTCL NOS and Gray zone lymphoma patients, the treatment was remarkably safe, with no dose-limiting toxicities or high-grade CRS or neurotoxicity [42]. Patients reported rapid recovery of physical function and a manageable symptom burden by one month post-infusion [42]. Even if results reported so far are mostly related to HL, the safety and efficacy of CD30 CAR-T have the potential to set this target in the TCL space as well.

### 1.8. Alternative Effector Platforms: The Promise of CAR-NK Cells

NK cells are innate effectors central to cancer immunosurveillance, killing targets through MHC-independent mechanisms. This biology makes them an attractive alternative to T-cells for CAR-directed therapy in PTCL, with several intrinsic advantages that address the principal failures of the autologous CAR-T platform. CAR-NK cells offer several intrinsic biological advantages that address the primary failures of CAR-T therapy.

Natural killer (NK) cells are components of the innate lymphoid compartment that contribute to immune-mediated tumor control through both CAR-dependent and CAR-independent mechanisms, utilizing natural receptors such as NKG2D, NKp30, and DNAM-1 [4,43]. This multi-modal killing may help prevent antigen escape, a common cause of relapse in CAR-T therapy [4,43].

From a safety perspective, CAR-NK cells are associated with a significantly lower risk of CRS and ICANS compared to CAR-T-cells, largely due to a different cytokine secretion profile, relying mainly on TNFα and IFNγ, and not Il-6 [43]. Furthermore, unlike allogeneic T-cells, NK cells do not induce GvHD, making them ideal candidates for “off-the-shelf” products derived from allogenic healthy donors, umbilical cord blood, or induced pluripotent stem cells (iPSCs) [4,44,45] (Table 3).

#### Engineering Challenges in CAR-NK Therapy

Despite their safety, CAR-NK cells face challenges related to low transduction efficiency and limited in vivo persistence [43]. To address these issues, newer generations of CAR-NK cells incorporate cytokine signaling motifs, such as IL-15, into the CAR construct or as a separate transgene to support cell survival without the need for exogenous cytokine administration [44].

In the context of T-cell malignancies, CD7-targeted CAR-NK cells have shown potent activity. However, because NK cells also express CD7, genomic disruption of the CD7 gene is still required to prevent NK-cell fratricide [46]. A study by Osorio-Rodríguez et al. successfully generated CD7-knockout CAR-NK cells from hPSCs, which demonstrated specific and robust cytotoxicity against T-ALL and PTCL cell lines while exhibiting normal expansion kinetics [47].

Although CD7 dominates the CAR-NK literature in this setting, the platform is not target-restricted. CD5-directed CAR-NK cells have shown preclinical activity against T-cell malignancies [48,49]. Additionally, CD4-directed CAR-NK approaches are relevant to CD4-expressing subtypes, AITL included, as well as a subset of PTCL-NOS [50]. Therefore, clonal-restricted TRBC1-directed NK constructs would, in principle, combine the aplasia-sparing selectivity of TRBC1 with the off-the-shelf, GvHD-free advantages of the NK platform, though they remain undeveloped. The recurring theme is that whichever antigen is chosen, if it is also expressed on NK cells (as for CD7); genomic disruption of that antigen in the effector remains necessary to prevent NK fratricide: the NK platform must be molded to suit the target biology.

### 1.9. Novel Perspectives: Rational Targets for the Next Generation

While CD5, CD7, and CD30 are the current frontrunners, several other antigens have emerged as rational targets based on the unique biology of T-cell malignancies. These targets are designed to minimize fratricide and improve the targeting of specific PTCL subtypes.

#### 1.9.1. CD37: Targeting Mature Lymphoid Antigens

CD37 is a tetraspanin glycoprotein expressed on mature B-cells and on subsets of cancerous T-cells in PTCL and cutaneous T-cell lymphoma (CTCL) [51]. This target is particularly attractive for the PTCL-NOS and CTCL populations where CD37 expression is most prevalent. Because CD37 is absent on T-cell progenitors and most resting T-cells, CD37-targeted CAR-T-cells do not exhibit significant fratricide [52]. Preclinical models have shown that anti-CD37 CARs can effectively eliminate PTCL cell lines and primary patient samples, leading to the initiation of a phase I trial (NCT04136275) [53]. This first-in-human phase 1 trial enrolled five patients with relapsed/refractory CD37+ lymphoid malignancies, which upon treatment achieved an 80% ORR, with three patients achieving complete responses [53]. Severe pancytopenia was the most notable toxicity occurring in three out of five patients.

CD19/CD37 dual CAR-T-cells are also being developed to address antigen escape, demonstrating superior tumor lysis against antigen-heterogeneous tumors compared to single-target CAR-T-cells in preclinical models, while combination strategies with MCL-1 inhibitors are explored to enhance efficacy in T-cell lymphomas in preclinical models [52,54].

#### 1.9.2. ROR1: Exploiting Epigenetic Dysregulation

The receptor tyrosine kinase-like orphan receptor 1 (ROR1) is an oncofetal antigen that is normally expressed during embryonic development and downregulated in adult tissues [55]. Its relevance to PTCL, however, is weak on present evidence: systematic immunophenotyping found T-cell non-Hodgkin lymphomas to be essentially ROR1-negative by immunohistochemistry (31 of 32 cases negative), unlike chronic lymphocytic leukemia [56]. Earlier proposals of ROR1 upregulation in a PTCL subset through loss of *TET2* are not corroborated at the protein level and require validation before ROR1 can be considered a rational PTCL target [57].

Because ROR1 is absent from healthy mature T-cells, a ROR1-directed approach would in principle avoid fratricide and T-cell aplasia; whether that theoretical advantage is relevant in PTCL depends, however, on an expression pattern that current data do not support. A phase I study of ROR1 CAR-T-cells (NCT02706392) enrolled 21 patients with chronic lymphocytic leukemia, triple-negative breast cancer and non-small-cell lung cancer—no T-cell lymphoma—and, while well tolerated, showed antitumor activity essentially confined to chronic lymphocytic leukemia, with responses in solid tumors limited by CAR immunogenicity and poor tumor infiltration; thus their efficacy in PTCL specifically remains entirely unestablished in clinical settings, and we present ROR1 as a speculative rather than a near-term target [58].

#### 1.9.3. CD70: Targeting the CD27-CD70 Axis

CD70, a member of the TNF superfamily, is typically expressed only on T, B, and NK cells, where its interaction with CD27 promotes cellular activation, proliferation, and differentiation [59]. CD70 is also aberrantly overexpressed in approximately 50% of PTCL cases, where it facilitates tumor growth and immune evasion, making it an attractive target for novel immune-mediated therapies, while the limited expression of CD70 on resting immune cells reduces the risk of long-term immunosuppression compared to pan-T targeting [59].

In the COBALT-LYM (NCT04502446), CD70 CAR-T-cells have shown promising results in patients with relapsed/refractory T-cell lymphoma, although CRS and ICANS represent a significant concern [60].

CD70-directed CAR-NK cells are also a promising “off-the-shelf” option for the treatment of T-cell malignancies. Preclinical models using cord-derived NK cells, engineered to exhibit a CD70-directed CAR construct as well as independent IL-15 secretion, show impressive cytotoxicity against TCL cell lines [61]. These results, consistent with the observation that CD70 expression is upregulated on alloreactive T-cells, support subsequent clinical translation, as the cytotoxic activity of all CD70 CAR-NK cells may be doubled by their potential to prevent GvHD in allogeneic settings by eliminating the recipient’s T-cells [62].

#### 1.9.4. CCR8: A Precision Target for Skin and Treg-Rich Malignancies

CCR8 is a chemokine receptor primarily expressed on skin-resident memory T-cells and tumor-infiltrating Tregs [12]. CCR8 is also highly expressed in CTCL and certain PTCL subtypes, particularly those with a Tfh origin or those exhibiting significant skin involvement [63].

Anti-CCR8 CAR-T-cells have demonstrated potent anti-tumor activity against ATLL and T-ALL cells in preclinical models [64]. The targeting of CCR8 is uniquely rational because it allows for the simultaneous eradication of the malignant T-cells and the depletion of the immunosuppressive Tregs within the TME, thereby “releasing the brakes” on the patient’s own immune system [65]. To this end, the development of dual CAR T-cells targeting CCR8 and CD7 may represent the future “gold standard” strategy to prevent antigen escape and overcome the immunosuppressive tumor microenvironment in both T-cell malignancies as well as solid tumors [64].

### 1.10. A Molecular-Subtype Framework for Rational Target Selection

In the preceding sections, we discuss potential individual antigenic CAR-T targets and provide evidence that collectively supports an approach that has yet to be fully translated into clinical practice: antigen selection for CAR-T therapy in PTCL should be individualized according to the molecular features of each disease subtype, rather than relying on a “one-size-fits-all” strategy. The GATA3-driven, Th2-polarized subset of PTCL-NOS, associated with inferior survival and a macrophage-rich microenvironment, co-expresses surface markers such as CCR4 [66]. These biological particularities make CCR4- or IL-4-receptor-directed approaches biologically rational in this specific subset, whereas the TBX21/Th1-cytotoxic subset more often retains CD7 and cytotoxic markers. AITL, a disease driven by TET2/RHOA-G17V alterations, can be targeted using the characteristic T-follicular helper phenotype (ICOS and CD10 expression). In ALCL, the near-constitutive expression of CD30 makes it a particularly attractive target, whereas EBV-associated ENKTL frequently expresses other actionable surface antigens, including CD38 and CD70. We stress the limits of this framework as forcefully as its promise: it is at present entirely hypothesis-generating, since no CAR-T trial has stratified enrolment or reported outcomes by molecular subtype, and the subtype-to-target correspondences rest on antigen-expression data and biological plausibility rather than comparative efficacy. The framework is nonetheless falsifiable as it predicts, for example, that CCR4-directed therapy should show subtype-dependent activity favoring GATA3+ disease, and that CD7-directed therapy will underperform in the CD7-low nodal cases identified in Section 2.1, and testing it requires trials that capture molecular subtype prospectively. Establishing subtype-stratified endpoints is, in our view, the single most important step toward moving CAR-T in PTCL from an empirical extension of B-cell strategies to genuinely precise cellular therapy.

Operationalizing this framework requires pretreatment stratification tools. These include molecular subtyping by immunohistochemistry or expression profiling to assign GATA3/TBX21 and follicular-helper differentiation. Additionally, considering the density-dependent CAR activity, as well as CD30 and CD7 heterogenous expression, quantitative and spatial assessment of target-antigen density are essential for target selection (Section 1.7 and Section 2.1). Equally important are the detection of circulating disease to determine the risk of apheresis-contamination (Section 2.2) together with ctDNA and minimal-residual-disease monitoring to identify emerging antigen-negative clones. None is yet validated as a predictive biomarker for CAR-T in PTCL, and building these readouts into trial eligibility and endpoints, rather than applying them post hoc, is the concrete prerequisite for biology-driven individualization.

While transcription factors like GATA3 are not directly targetable by traditional CAR designs, the PTCL-GATA3 subtype’s high reliance on Th2 signaling pathways suggests that CARs targeting downstream or associated surface markers, such as CCR4 or IL-4 receptor, may be highly effective in this specific cohort [19]. The use of GATA3 as a biomarker to select patients for specific surface-antigen-targeted CARs represents a move toward more personalized immunotherapy in the TCL space [17].

## 2. Lineage-Specific Barriers That Temper the Current Evidence in PTCL

The engineering advances that address fratricide (Section 1.3) have largely solved a manufacturing problem, but they do not resolve the biological and clinical obstacles specific to the T-cell lineage and to mature PTCL in particular. Several of these barriers are under-represented in the current literature because the pivotal efficacy data derive from lymphoblastic disease, where they are less pronounced. We consider them here explicitly, as they determine whether, and in whom, CAR-based therapy is presently feasible.

### 2.1. Antigen Loss and Heterogeneity as a Pre-Existing Route to Escape

Antigen-negative relapse, the paradigmatic resistance mechanism of CD19-directed therapy, is a particular concern in mature PTCL, where the target antigens are frequently downregulated at baseline rather than lost only under selective pressure. As noted above, CD7 is often reduced or absent in nodal PTCL [26] and CD5 is heterogeneously expressed and rapidly internalized upon CAR engagement (Section 1.5). A partially antigen-negative malignant population may therefore pre-exist treatment, providing an immediate reservoir for treatment escape rather than emerging only as a result of treatment exposure. Antigen expression should thus be treated as a patient-selection criterion, not an assumption, and single-antigen targeting is intrinsically vulnerable in a disease this heterogeneous, sustaining the principal rationale for the dual-target and logic-gated designs discussed in Section 3.

### 2.2. Leukemic and Circulating Disease: The Excluded Patients Who Most Need Therapy

A substantial fraction of T-cell malignancies present with circulating disease (e.g., Sézary syndrome, adult T-cell leukemia/lymphoma, T-prolymphocytic leukemia, hepatosplenic T-cell lymphoma and a subset of PTCL). In autologous platforms, circulating malignant cells cannot be reliably separated from normal T-cells during apheresis, risking product contamination and inadvertent transduction of the tumor clone [67]. Such patients are consequently excluded from many CAR-T trials, yet they often represent the aggressive, therapy-refractory populations with the greatest unmet need. This is a structural selection bias in the existing evidence: reported response rates are generated in the patients most amenable to manufacturing, not necessarily in those for whom the therapy is most needed.

### 2.3. Apheresis-Limited Lymphopenia and the Case for Allogeneic Platforms

Patients with relapsed/refractory PTCL are typically lymphopenic from disease burden and prior lymphotoxic therapy, which compromises the feasibility and quality of autologous manufacturing through insufficient apheresis yield, suboptimal T-cell fitness and manufacturing failure. This constraint is one of the strongest arguments for the allogeneic, off-the-shelf platforms favored throughout this review, which decouple the therapy from the patient’s exhausted T-cell compartment. The argument is not unqualified: allogeneic T-cell products require additional TCR disruption to prevent GvHD, may show limited persistence and immune-mediated rejection, and, as the fatal EBV-driven DLBCL in the RD13-01 trial illustrates (Section 1.4), carry the risk of profound, prolonged immunosuppression that can itself be lethal. The allogeneic route relocates the problem rather than eliminating it.

### 2.4. Lymphodepletion: An Under-Recognized Contributor to T-Cell Aplasia

Almost every trial cited here uses fludarabine/cyclophosphamide conditioning carried over unmodified from B-cell CAR-T, with no T-cell-specific optimization; even the PTCL-oriented NS7CAR-T study [68] employed standard fludarabine 30 mg/m^2^/day and cyclophosphamide 300 mg/m^2^/day for three days (Table 2). This matters mechanistically, because fludarabine is itself durably T-lymphotoxic: the profound and prolonged T-cell aplasia routinely attributed to on-target CAR activity is more accurately a composite of baseline disease- and therapy-related lymphopenia, lymphodepleting conditioning and on-target depletion. Comparisons of the safety margins of different targets (for example CD5 versus CD7) should therefore be made cautiously when the conditioning backbone that co-determines that toxicity is essentially identical and unoptimized across trials. Conditioning is a modifiable variable that has received almost no dedicated study in the T-cell setting, and this is a concrete future direction.

### 2.5. Extrapolation from Lymphoblastic Disease and Dependence on Transplant

Two caveats bound the field. First, as established in Section 1.4, the most impressive response figures are generated in T-ALL/T-LBL and are not transferable to mature PTCL, whose dataset remains early, small and short in follow-up. Second, for the pan-T targets, consolidative allogeneic HSCT is frequently required after remission to reconstitute immunity and forestall antigen-negative relapse [69]. Much of CD7-directed therapy therefore functions, at present, as a bridge to transplant rather than as definitive treatment, a positioning we make explicit because it materially changes how these therapies compare with existing salvage and transplant strategies. A practical corollary concerns bridging therapy in the manufacturing window: because autologous production takes weeks and relapsed/refractory PTCL can progress rapidly, disease control between apheresis and infusion is often necessary, and radiotherapy, established as a bridging modality in B-cell CAR-T, ref. [70], is a rational option for anatomically limited or symptomatic sites, although the frequently disseminated presentation of PTCL limits localized approaches and T-cell-specific data remain scarce.

## 3. Enhancing Persistence: Overcoming Resistance vs. Long-Term Safety

The durability of response in PTCL remains a critical concern, with some trials reporting a high incidence of early relapse due to antigen escape or T-cell exhaustion [4].

To combat the immunosuppressive TME, researchers are developing “armored” CARs that secrete pro-inflammatory cytokines such as IL-12 or IL-18, or incorporate costimulatory domains that enhance persistence, such as 4-1BB or modified CD28 [4,44,71]. Furthermore, the use of logic-gated CARs—such as those utilizing synNotch receptors or “AND-gate” logic—can improve specificity by requiring the presence of two antigens on the tumor cell surface, thereby reducing the risk of on-target, off-tumor toxicity on healthy tissues expressing a single marker [4].

The integration of CRISPR/Cas9 and other gene-editing tools into the CAR-T manufacturing process has raised concerns about off-target effects and genomic stability [72]. While the FDA reported 22 cases of T-cell malignancies following CAR-T therapy, the majority of these were not derived from CAR-positive cells, and the absolute risk remains very low [73]. Nevertheless, the use of site-specific integration methods, such as targeting the CAR to the *TRAC* locus, may offer a safer and more uniform alternative to traditional lentiviral transduction by reducing the risk of insertional mutagenesis [72].

## 4. Conclusions and Perspective

**Figure 4:**
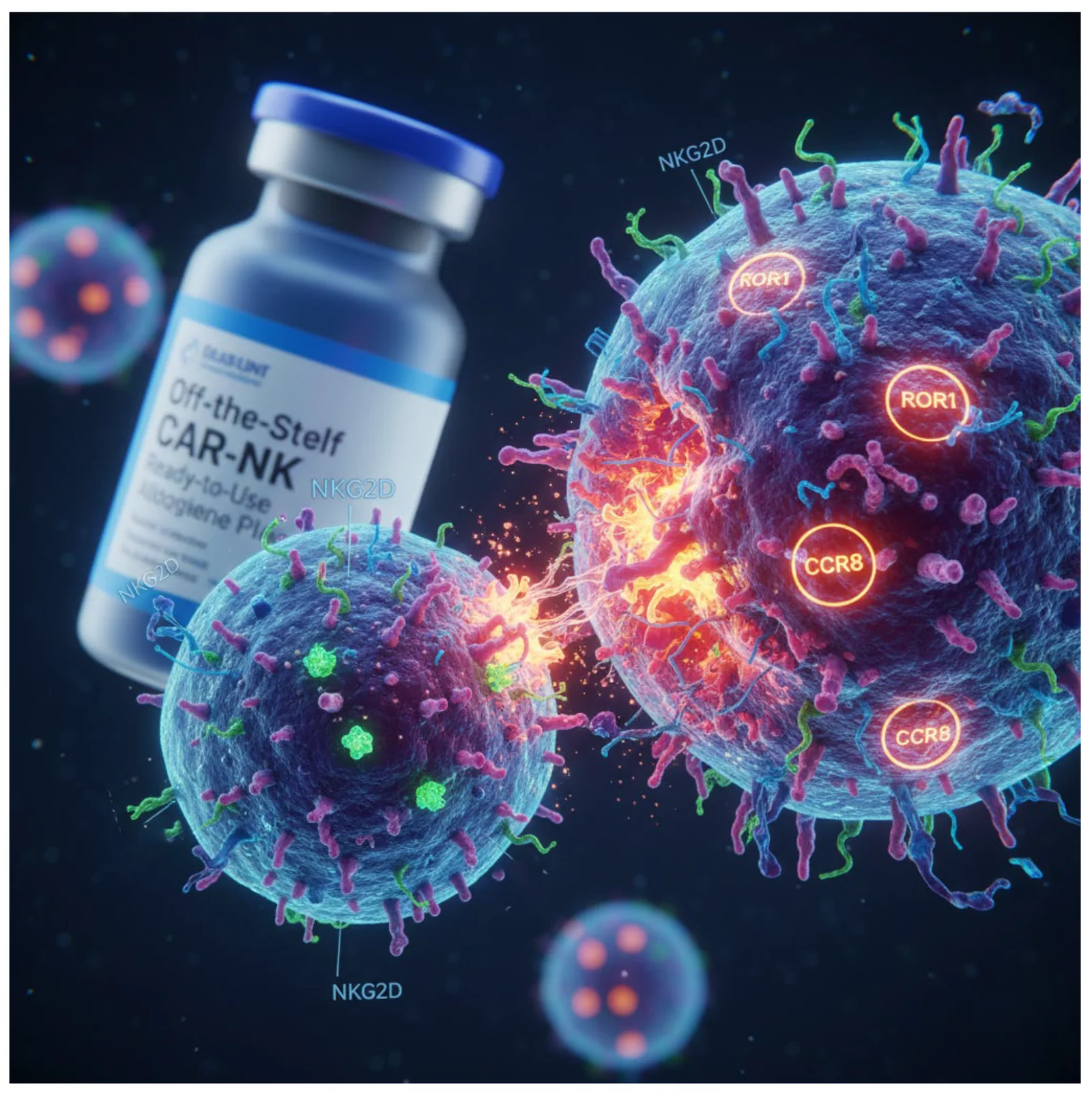
Next-generation effector platforms and novel antigens. The transition from autologous T-cells to allogeneic “off-the-shelf” CAR-NK cells and iPSC-derived platforms. This figure showcases the intrinsic advantages of NK cells—including lower CRS/ICANS risk and multiple killing mechanisms (NKG2D, DNAM-1)—and highlights emerging “fratricide-free” targets like ROR1, CD37, and CCR8.

CAR-based therapy in PTCL has moved from a manufacturing impasse toward early clinical signals whose significance should not be overstated (Figure 4). The technical challenges of fratricide and product contamination with malignant cells have been largely solved through gene editing and alternative effector platforms; T-cell aplasia, antigen escape and dependence on consolidative transplant have not. The most mature response data derive predominantly from lymphoblastic disease, while the mature-PTCL dataset remains small and drawn from selected patients, and for the pan-T targets much of the field presently functions as a bridge to allogeneic HSCT rather than as definitive therapy.

The clearest path forward is not a new construct or target but a change in trial design—capturing molecular subtype, antigen density and circulating disease prospectively—so that target selection becomes genuinely biology-driven across the broader treatment landscape, whether as a bridge to allogeneic HSCT or as a standalone option. Only subtype-stratified evidence will establish whether these therapies can achieve, in PTCL, the transformation they produced in B-cell malignancies.

## Figures and Tables

**Table 1:** Overview of common surface antigen expression, key molecular drivers, and potential CAR T-cell targets across major peripheral T-cell lymphoma subtypes.

Malignancy Subtype	Common Surface Antigens	Molecular Drivers	CAR Targeting Priority
PTCL-NOS (GATA3+) [15]	CD3, CD4, CD5, CD7, CCR4	GATA3, STAT3	CD5, CD7, CCR4
PTCL-NOS (TBX21+) [15]	CD3, CD8, CD7, CD30 (subset)	TBX21, CXCR3	CD7, CD30 (17)
AITL (Tfh origin) [9]	CD3, CD4, CD5, CD10, ICOS	TET2, RHOA, DNMT3A	CD5, CD7
ALCL (ALK +/−) [9]	CD30, CD25, CD2, CD4	ALK rearrangements, STAT3	CD30 (Primary)
ENKTL (EBV+) [11]	CD2, CD56, CD38, CD70	EBV-related pathways	CD38, CD70, LMP1 (20)

**Table 2:** Summary of main clinical CAR-T clinical trials developed in the field of T cell malignancies.

Trial/Product	Target Population	Response (CR)	Key Safety Findings	Persistence Strategy
RD13-01 [30]	PTCL, T-ALL, AML	63.6%	No GVHD; viral reactivation risk	Allogeneic (Donor-derived)
NS7 CAR-T [31]	T-ALL/LBL	94.4%	Low-grade CRS (90%); 5% ICANS	Autologous (PEBL-modified)
PA3-17 [32]	T-ALL/LBL	90%	High-grade cytopenias	CRISPR-mediated CD7 KO

**Table 3:** Main types of cellular therapy and main tocixity profile.

Effector Cell Type	GvHD Risk	CRS/ICANS Risk	Manufacturing	Persistence
Autologous CAR-T [4]	Minimal	High	Slow, complex	Long-term
Allogeneic CAR-T [5]	High (requires KO)	High	Rapid (off-the-shelf)	Variable
CAR-NK Cells [45]	Low/Absent	Low	Rapid (off-the-shelf)	Short-term

## Data Availability

No new data were created or analyzed in this study. Data sharing is not applicable to this article.
